# Development of a Radioiodinated Triazolopyrimidine Probe for Nuclear Medical Imaging of Fatty Acid Binding Protein 4

**DOI:** 10.1371/journal.pone.0094668

**Published:** 2014-04-14

**Authors:** Kantaro Nishigori, Takashi Temma, Satoru Onoe, Sotaro Sampei, Ikuo Kimura, Masahiro Ono, Hideo Saji

**Affiliations:** 1 Department of Patho-Functional Bioanalysis, Graduate School of Pharmaceutical Sciences, Kyoto University, Sakyo-ku, Kyoto, Japan; 2 Department of Applied Biological Science, Graduate School of Agriculture, Tokyo University of Agriculture and Technology, Fuchu-Shi, Tokyo, Japan; 3 Department of Pharmacogenomics, Graduate School of Pharmaceutical Sciences, Kyoto University, Sakyo-ku, Kyoto, Japan; Genentech, United States of America

## Abstract

Fatty acid binding protein 4 (FABP4) is the most well-characterized FABP isoform. FABP4 regulates inflammatory pathways in adipocytes and macrophages and is involved in both inflammatory diseases and tumor formation. FABP4 expression was recently reported for glioblastoma, where it may participate in disease malignancy. While FABP4 is a potential molecular imaging target, with the exception of a tritium labeled probe there are no reports of other nuclear imaging probes that target this protein. Here we designed and synthesized a nuclear imaging probe, [^123^I]TAP1, and evaluated its potential as a FABP4 targeting probe in *in vitro* and *in vivo* assays. We focused on the unique structure of a triazolopyrimidine scaffold that lacks a carboxylic acid to design the TAP1 probe that can undergo facilitated delivery across cell membranes. The affinity of synthesized TAP1 was measured using FABP4 and 8-anilino-1-naphthalene sulfonic acid. [^125^I]TAP1 was synthesized by iododestannylation of a precursor, followed by affinity and selectivity measurements using immobilized FABPs. Biodistributions in normal and C6 glioblastoma-bearing mice were evaluated, and excised tumors were subjected to autoradiography and immunohistochemistry. TAP1 and [^125^I]TAP1 showed high affinity for FABP4 (*K*_i_ = 44.5±9.8 nM, *K*_d_ = 69.1±12.3 nM). The FABP4 binding affinity of [^125^I]TAP1 was 11.5- and 35.5-fold higher than for FABP3 and FABP5, respectively. In an *in vivo* study [^125^I]TAP1 displayed high stability against deiodination and degradation, and moderate radioactivity accumulation in C6 tumors (1.37±0.24% dose/g 3 hr after injection). The radioactivity distribution profile in tumors partially corresponded to the FABP4 positive area and was also affected by perfusion. The results indicate that [^125^I]TAP1 could detect FABP4 *in vitro* and partly *in vivo*. As such, [^125^I]TAP1 is a promising lead compound for further refinement for use in *in vivo* FABP4 imaging.

## Introduction

Fatty acid binding proteins (FABPs), a group of proteins that regulate lipid responses in cells, are known to be involved in metabolic and inflammatory pathways [1]. Among their various functions, FABPs transport lipids to specific cell components such as lipid droplets, the endoplasmic reticulum, and mitochondria [1]. Through this lipid transport, FABPs regulate lipid usage in cells for storage, signaling, membrane synthesis, oxidation, and transcriptional regulation.

FABP4 (also known as Adipocyte FABP) is the best-characterized isoform among the FABPs. FABP4 is predominantly expressed in adipocytes and macrophages [1] where it regulates the activities of Jun *N*-terminal kinase (JNK) 1 and hormone-sensitive lipase (HSL) [2], [3]. FABP4 suppresses the PI3K/Akt signaling pathway that includes the insulin receptor β, which reduces insulin sensitivity as well as dyslipidemia and insulin resistance [4], [5]. In addition, FABP4 activates JNK1 to enhance production of cytokines such as tumor necrosis factor α (TNF-α), interleukin 1β, and monocyte chemoattractant protein 1 (MCP-1), which in turn propagates inflammatory reactions in arthritis, atherosclerosis, non-insulin-dependent diabetes mellitus and tumor formation [6].

Recently a relationship between FABP4 expression and glioma malignancy was reported [7]. Compared to normal brain tissue and lower-grade glial tumors, in human glioblastoma lesions (GBM, grade 4 glioma) FABP4 was reportedly expressed at significantly higher levels in phagocytic macrophage-like cells (or presumably premature microglias) and microvascular endothelial cells. Although the detailed functions of FABP4 in GBM remain unclear, they are thought to be related to angiogenesis that is associated with GBM formation. Therefore, FABP4 is a potential molecular imaging target for evaluating glioma malignancy, for which there are currently few nuclear imaging tools beyond a tritium labeled probe [8]. Therefore, in this study we aimed to develop a novel radiolabeled-probe to detect FABP4. This FABP4-targeting probe, which is suitable for single photon emission computed tomography (SPECT), may have valuable applications for evaluating glioma malignancies, elucidating the function of FABP4 and the development of therapeutic agents that target FABP4.

Recent studies have reported a variety of low molecular weight FABP4 inhibitors such as BMS309403 [9] and HTS01037 [10] that have high FABP4 affinity and high selectivity over other FABP isoforms. These inhibitors are currently being developed as effective drugs for the treatment of inflammatory diseases. Indeed, Furuhashi *et al.* demonstrated that BMS309403 treatment improved glucose metabolism and enhanced insulin sensitivity in a diabetes mouse model and reduced atherosclerotic lesions in an arteriosclerosis mouse model [4]. Another report from Lan *et al.* [11] showed that triazolopyrimidine-based compounds synthesized as novel FABP4 inhibitors had high affinity and high selectivity for FABP4. These compounds in particular are unique in their lack of non-carboxylic acid structures, which would be desirable as a core structure in imaging probes that target intracellular molecules after facilitated diffusion across cell membranes. Taking into account these considerations, we designed a nuclear imaging probe, [^123^I]TAP1, which is based on the triazolopyrimidine scaffold. We first synthesized [^125^I]TAP1 and investigated its specificity and selectivity for FABP4 and its physicochemical characteristics *in vitro*. Next, we evaluated the *in vivo* capacity of [^125^I]TAP1 to recognize FABP4 in normal and glioblastoma-bearing mice.

## Materials and Methods

### 1. General

All reagents were purchased from Nacalai Tesque, Inc. and Wakenyaku Co., Ltd. and were used without further purification unless otherwise noted. 1H-NMR spectra were obtained at 400 MHz on JEOL JNM-AL400 NMR spectrometers at room temperature with tetramethylsilane (TMS) as an internal standard. Chemical shifts are reported as δ values (parts per million) relative to the TMS standard. Coupling constants are reported in Hertz. Multiplicity is defined by s (singlet), d (doublet), t (triplet), and m (multiplet). High resolution mass spectra (HRMS) were acquired on a JMS-SX 102A QQ or JMS-GC-mate mass spectrometer (JEOL). Recombinant hexahistidine (his)-tagged FABP3, FABP4 and FABP5 proteins were purchased from Cayman Chemical Company.

### 2. Animals

Animal experiments were conducted in accordance with our institutional guidelines and were approved by the Kyoto University Animal Care Committee (Permit Number: 2012-49, 2013-33). Male ddY mice, male Balb/c nu-nu mice and male Sprague-Dawley rats were supplied by Japan SLC, Inc. Animals were fed standard chow and had access to water *ad libitum*. All surgery was performed under isoflurane anesthesia, and all efforts were made to minimize suffering.

### 3. Cell culture

Human monocytic leukemia THP-1 cells were obtained from ATCC and rat glioma C6 cells were from the Health Science Research Resources Bank. THP-1 cells were cultured in Gibco RPMI 1640 medium (Nissui Pharmaceutical Co., Ltd.) supplemented with 10% heat-inactivated fetal bovine serum (Nichirei Bioscience Inc.), 50 U/ml penicillin and 50 µg/ml streptomycin (Nacalai Tesque, Inc.) at 37 °C in 5% CO_2_. C6 cells were cultured in McCoy's 5A Modified Medium supplemented with 10% heat-inactivated fetal bovine serum, 50 U/ml penicillin and 50 µg/ml streptomycin at 37 °C in 5% CO_2_. For cell uptake assays, THP-1 monocytes were differentiated into macrophages by incubation with 100 nM phorbol 12-myristate 13-acetate for 24 hr.

### 4. Preparation of tumor-bearing mice

C6 cells were suspended in PBS(-) followed by subcutaneous inoculation into the right hind legs of Balb/c nu-nu mice (5×10^6^ cells/100 µL).

### 5. Chemistry

5-(Chloromethyl)-2-phenyl-[1], [2], [4]triazolo[1,5-a]pyrimidin-7(4*H*)-one (1)

Compound **1** was synthesized according to previously reported procedures [12], [13].

5-{(3-Iodophenoxy)methyl}-2-phenyl-[1], [2], [4]triazolo[1,5-a]pyrimidin-7(4*H*)-one (2)

K_2_CO_3_ (0.21 g, 1.53 mmol) was added to a solution of 3-iodophenol (0.34 g, 1.53 mmol) in 7 ml DMF, and the mixture was stirred for 30 min at 80 °C. Then **1** (0.20 g, 0.77 mmol) in 3 ml DMF was added to the reaction mixture, which was stirred for 19 hr at 85°C. The solvent was then evaporated under reduced pressure, water was added, and the product extracted into ethyl acetate. The organic layer was dried over Na_2_SO_4_, filtered, the solvent was evaporated, and the residue purified by silica gel chromatography (CHCl_3_/methanol = 10/1) to yield **2** (non-radioactive TAP1, 0.24 mmol, 15.7% yield). Analytical data for **2** are as follows: 1H NMR (DMSO-d_6_, 400 MHz): 8.12–8.10 (m, 2H), 7.54–7.46 (m, 3H), 7.43–7.42 (t, 1H), 7.36–7.33 (dt, 1H), 7.13–7.06 (m, 2H), 5.96 (s, 1H), 5.05 (s, 2H). 13C NMR (DMSO-d6, 100 MHz): δ65.5, 95.1, 98.1, 114.7, 123.6, 126.6, 128.9, 129.9, 130.4, 130.5, 131.5, 151.6, 155.8, 158.1. HRMS (FAB+): m/z calcd for C_18_H_14_IN_4_O_2_ 445.0162, found 445.0160.

5-{(3-Bromophenoxy)methyl}-2-phenyl-[1], [2], [4]triazolo[1,5-a]pyrimidin-7(4*H*)-one (3)

K_2_CO_3_ (0.32 g, 2.31 mmol) was added to a solution of 3-bromophenol (0.27 g, 1.53 mmol) in 7 ml DMF, and the mixture was stirred for 30 min at 80 °C. Then **1** (0.20 g, 0.77 mmol) in 3 ml DMF was added to the reaction mixture, and the reaction was stirred for 7 hr at 85°C. The solvent was then evaporated under reduced pressure, water was added, and the product extracted into ethyl acetate. The organic layer was dried over Na_2_SO_4_, filtered, the solvent evaporated, and the residue purified by silica gel chromatography (CHCl_3_/methanol = 10/1) to give **3** (0.15 mmol, 9.8% yield). Analytical data for **3** are as follows: 1H NMR (DMSO-d_6_, 400 MHz): 8.12–8.10 (d, 2H), 7.52–7.51 (m, 3H), 7.30–7.27 (m, 2H), 7.20–7.18 (m, 1H), 7.08–7.06 (m, 1H), 6.03 (s, 1H), 5.10 (s, 2H). 13C NMR (DMSO-d6, 100 MHz): δ66.3, 97.4, 114.3, 117.9, 122.1, 124.4, 126.6, 128.9, 130.2, 130.3, 131.3, 151.1, 152.6, 156.1, 158.5, 160.6. HRMS (FAB+): m/z calcd for C_18_H_14_BrN_4_O_2_ 397.0300, found 397.0295.

2-Phenyl-5-[{3-(tributylstannyl)phenoxy}methyl][1], [2], [4]triazolo[1,5-a]pyrimidin-7(4*H*)-one (4)

A mixture of **3** (0.11 g, 0.27 mmol), bis(tributyltin) (0.31 g, 0.54 mmol), and (Ph_3_P)_4_Pd (0.031 g, 0.027 mmol) in a mixed solvent of 5.0 ml of triethylamine and 10 ml DMF was stirred for 4 hr with reflux. The solvent was then evaporated under reduced pressure, and the residue was purified by silica gel chromatography (CHCl_3_/methanol = 10/1) to give **4** (TAP1 precursor, 0.042 mmol, 15.6% yield). Analytical data for **4** are as follows: 1H NMR (CDCl_3_, 400 MHz): 8.23–8.21 (m, 2H), 7.47–7.45 (m, 3H), 7.29–7.26 (m, 1H), 7.15–7.14 (d, 1H), 7.04–7.03 (d, 1H), 6.79–6.76 (s, 1H), 6.11 (s, 1H), 5.06 (s, 2H), 1.58–1.50 (m, 6H), 1.38–1.25 (m, 6H), 1.09–1.05 (m, 6H), 0.91–0.87 (m, 9H). 13C NMR (CDCl_3_, 100 MHz): δ9.6, 13.7, 27.3, 29.0, 64.8, 98.2, 113.3, 122.4, 127.4, 128.7, 129.0, 129.2, 130.5, 130.8, 144.6, 148.8, 150.7, 156.1, 156.2, 162.1. HRMS (FAB+): m/z calcd for C_30_H_41_N_4_O_2_Sn 609.2252 found 609.2245.

### 6. *In vitro* study of TAP1

#### 6.1. Binding assay

In accordance with previous reports, competition binding experiments were performed using 8-anilino-1-naphthalene sulfonic acid (1,8-ANS) as the tracer. Briefly, a mixture containing 0.12 ml phosphate buffer (50 mM, pH = 7.4), 0.03 ml TAP1 (2.6 mM–300 nM) in DMSO, 0.075 ml 1,8-ANS (24 nM) in phosphate buffer (0.2% ethanol, v/v) and 0.075 ml his-tagged FABP4 (1 µM) in phosphate buffer was incubated at room temperature for 5 min. The fluorescence intensity at an excitation and emission wavelength of 370 and 475 nm, respectively, was plotted, and values for the half-maximal inhibitory concentration (IC_50_) were determined from displacement curves of three independent experiments using GraphPad Software (GraphPad Software, San Diego, CA). The inhibition constants (*K*_i_) were calculated using the Cheng-Prusoff equation: *K*_i_ = IC_50_/(1+[L]/*K*_d_), where [L] is the 1,8-ANS concentration and *K*_d_ is the 1,8-ANS dissociation constant. The *K*_d_ value of 1,8-ANS for FABP4 was 1.2 µM.

#### 6.2. Parallel artificial membrane permeation assay (PAMPA)

The permeability of TAP1 was measured using a pre-coated PAMPA plate system (BD Biosciences) following the manufacturer's procedures. Briefly, TAP1 (100–200 µM, 0.2 ml) was added to donor plate and PBS (-) (0.2 ml) was added to the acceptor plate in the PAMPA plate assembly. After coupling both plates together and incubating at room temperature for 5 hr, 0.15 ml of the sample was obtained from each plate and 0.15 ml of the original compound solution was also obtained. Then, the absorbance of each sample was measured at 280 nm. The permeability was calculated as: Pe (cm/s) = (−ln[1−C_A_/C_equilibrium_])/St (1/V_D_+1/V_A_) and C_equilibrium_ = [C_D_V_D_+C_A_V_A_]/(V_D_+V_A_). C_D_ = C_0_ (A_D_−A_buffer_)/(A_0_−A_buffer_) and C_A_ = C_0_ (A_A_−A_buffer_)/(A_0_−A_buffer_). C_0_ is the concentration of the start sample and A_0_, A_buffer_, A_D_ and A_A_ are absorbances of the starting sample, buffer, donor and acceptor, respectively. V_D_ and V_A_ are the donor and acceptor volumes, respectively. S is the membrane area and t is the incubation time.

### 7. Radiosynthesis

[^125^I]TAP1 was prepared from the tributyltin precursor by iododestannylation. Briefly, 0.02 ml [^125^I]NaI (37 MBq, specific activity 81.4 TBq/mmol, MP Biomedicals) was added to a mixture of 0.044 ml *N*-chlorosuccinimide (NCS) in methanol (0.5 mg/ml) and 0.163 ml of **5** in methanol (1% acetic acid, v/v, 4.0 mg/ml). The reaction was allowed to proceed at room temperature for 30 min and terminated by addition of 0.01 ml saturated aqueous NaHSO_3_. After solvent removal under a nitrogen gas stream, the residue was extracted with ethyl acetate. The extract was dried by passage through an anhydrous Na_2_SO_4_ column, and the solvent removed with a stream of nitrogen gas. [^125^I]TAP1 was purified by RP-HPLC on a Cosmosil C18 column, eluting with an isocratic solvent of H_2_O (0.1% TFA)/acetonitrile (0.1% TFA) (37/63) at a flow rate of 1.5 ml/min.

### 8. *In vitro* study of [^125^I]TAP1

#### 8.1. Binding assay

For the selectivity binding assay, his-tagged FABP3 (0.75 mg/ml), FABP4 (0.75 mg/ml) and FABP5 (0.70 mg/ml) in 50 mM phosphate buffer containing 100 mM NaCl (20% glycerol, v/v, pH = 7.2) were used. Immobilization was performed following the manufacturer's procedures. Each solution of his-tagged FABP3 (0.003 ml, 1.5 µg), FABP4 (0.002 ml, 1.5 µg) or FABP5 (0.002 ml, 1.4 µg) was incubated with 0.02 ml Ni-NTA Magnetic Agarose Beads (Qiagen) and 0.5 ml protein binding buffer (50 mM NaH_2_PO_4_, 300 mM NaCl, 10 mM imidazole, pH = 8.0) at room temperature for 1 hr. After supernatant removal, protein binding buffer with 1% BSA was added, and the mixture was incubated at room temperature for 30 min. After removal of the supernatant, 0.4 ml of interaction buffer (50 mM NaH_2_PO_4_, 300 mM NaCl, 10 mM imidazole, and 0.005% Tween, v/v, pH = 8.0) and 0.05 ml [^125^I]TAP1 (0.01 MBq) in interaction buffer (5% ethanol, v/v) were added. For measurement of non-specific binding, 0.05 ml of nonradioactive TAP1 in interaction buffer (5% ethanol, v/v, 11.3 µM) was added with the [^125^I]TAP1. After incubation at room temperature for 2 hr, the supernatants were removed, and the beads washed with interaction buffer (5% ethanol, v/v). The radioactivity of beads in the tubes was measured with a well-type γ-counter (1480 Wizard3, PerkinElmer Japan Co., Osaka, Japan). Binding ratios were calculated as (radioactivity of beads)/(total applied radioactivity)/(protein abundance on beads)×100 (%Dose/µg protein).

For the saturation assay, a mixture solution of [^125^I]TAP1 (0.3–9.5 MBq) was prepared by mixing nonradioactive TAP1 (0.0125–0.4 µM) in interaction buffer (2.5% ethanol and 5% DMSO, v/v). Nonspecific binding was defined in the presence of 0.09 mg/ml [^125^I]TAP1 including nonradioactive TAP1. After FABP4 immobilization as described above, 0.4 ml interaction buffer and 0.05 ml mixture solution were added, and the mixture was incubated at room temperature for 2 hr. After incubation, the supernatants were removed and the beads were washed with interaction buffer (5**%** ethanol, v/v). The radioactivity of beads in the tubes was then measured with a well-type γ-counter. The dissociation constant (*K*_d_) of [^125^I]TAP1 for FABP4 was determined by Scatchard analysis using GraphPad Prism.

#### 8.2. Cell uptake study

For the cell uptake study, tubes were pre-incubated with 50 mg/ml skim milk in PBS (-) at 4 °C for 3 hr. Then, 0.4 ml of differentiated THP-1 cells (1.0×10^6^ cells/tube) in PBS (-) and 0.05 ml [^125^I]TAP1 in PBS (-) (0.008 MBq, 5% ethanol, v/v) were added to the tubes, and the cells were incubated at 4 °C for 3 hr. After centrifugation (4,160×*g*, 5 min), the supernatants were removed, and the cells were washed twice with PBS (-) (0.5% ethanol, v/v). The radioactivity of the tubes was measured with a well-type γ-counter. After the cells were lysed with 0.25 ml 2 N NaOH, protein determination was performed. Binding ratios were calculated as (radioactivity in tubes)/(total applied radioactivity)/(protein abundance) ×100 (%Dose/mg protein). For the inhibition assay with nonradioactive TAP1 or BMS309403, 0.05 ml nonradioactive TAP1 in PBS(-) (500–0.01 µM, 10% DMSO, v/v) or 0.05 ml BMS309403 in PBS (-) (500 µM, 10% DMSO, v/v) was added to a mixture of 0.4 ml differentiated THP-1 cells (1.0×10^6^ cells/tube) and 0.05 ml [^125^I]TAP1 (0.008 MBq). After incubation at 4 °C for 3 hr, the radioactivity measurement and calculation of the binding ratio were performed as described above. Data are presented as the ratio relative to the non-inhibitor group. In addition, a cell uptake study using 3T3-L1 preadipocytes and differentiated adipocytes was similarly performed (the detailed method is described in Supporting Information).

#### 8.3. Determination of the partition coefficient, stability, and serum protein binding

Experimental determination of the [^125^I]TAP1 partition coefficient was performed with 1-octanol and phosphate buffer at pH 7.4. The two phases were pre-saturated, and a mixture solution of 3.0 ml 1-octanol and 3.0 ml PBS (-) was added to a tube containing 0.02 ml [^125^I]TAP1 in PBS (-) (0.024 MBq, 5% ethanol, v/v). The test tube was vortexed and centrifuged (1,000×*g*, 5 min). Aliquots from the 1-octanol and buffer phases (0.5 ml) were counted with a well-type γ-counter. This procedure was repeated three times. The partition coefficient was calculated from the ratio of radioactivity in the organic and aqueous layers.

The *in vitro* stability of [^125^I]TAP1 in plasma was measured using plasma samples taken from ddY mice (males, 7 weeks old). [^125^I]TAP1 (0.07 MBq, 0.01 ml) was incubated with 0.1 ml plasma at 37 °C for 2 hr. After addition of 0.2 ml methanol and vortexing, the mixture was centrifuged (4,160×*g*, 10 min). The supernatant was filtered with a low protein binding hydrophilic PTFE membrane (Millex-LH MILLIPORE), and the filtrate was analyzed by RP-HPLC using a Cosmosil C18 column with an isocratic solvent of H_2_O (0.1% TFA, v/v)/acetonitrile (0.1% TFA, v/v) (37/63) at a flow rate of 1.5 ml/min.

The serum protein binding ratio of [^125^I]TAP1 was measured using rapid equilibrium dialysis (RED) devices (Thermo Fisher Scientific K.K.) following the manufacturer's procedures. Briefly, [^125^I]TAP1 (0.02 MBq, 0.02 ml) and mouse plasma (0.3 ml) were mixed and placed to the sample chamber and 0.5 ml PBS (-) was placed in the RED device buffer chamber. After incubation at 37 °C for 4 hr, 0.05 ml of the samples were obtained from each chamber. The binding ratios were calculated as: (sample chamber radioactivity)-(buffer chamber radioactivity)/(sample chamber radioactivity) x 100 (%).

### 9. *In vivo* and *ex vivo* studies using [^125^I]TAP1

#### 9.1. Biodistribution in normal mice

A saline solution (5% ethanol, v/v) of [^125^I]TAP1 (0.035 MBq) was injected intravenously into the tail vein of ddY mice (males, 5 weeks old). The mice were sacrificed at various post-injection time points, and the organs of interest were removed. The organ weights were determined, and the radioactivity in the organs was measured with a well-type γ-counter.

#### 9.2. Metabolite analysis

A saline solution (5% ethanol, v/v) of [^125^I]TAP1 (1.20 MBq) was injected intravenously into the tail vein of ddY mice (males, 5 weeks old), and animals were sacrificed 1 hr post-injection. The liver and kidneys were removed and homogenized in 1 ml ice-cold 30 mM Tris-HCl buffer (pH = 8.5) containing 5 mM magnesium acetate at 4 °C. After addition of 3 ml cold methanol and vortexing, tissue samples were centrifuged (10,000×*g*, 5 min). The supernatants were filtered, and the filtrate was analyzed by RP-HPLC as described above.

#### 9.3. Biodistribution in C6 bearing mice

A saline solution (5% ethanol, v/v) of [^125^I]TAP1 (0.06 MBq) was injected intravenously into the tail vein of C6 cell implantation model Balb/c nu-nu mice (males, 8 weeks old). The mice were sacrificed at various post-injection time points and organs, including tumor tissues, were removed. The weight and radioactivity of the organs were measured as mentioned above. Several model mice were sacrificed 3 hr after the injection by transcardial perfusion of saline (20 ml) under anesthesia with chloral hydrate. The weight and radioactivity of the removed organs, including tumor tissues, were measured as mentioned above.

#### 9.4. *Ex vivo* autoradiography

Tumor-bearing mice were sacrificed 3 h after intravenous administration of [^125^I]TAP1 (0.47 MBq). The tumors were removed and immediately frozen. Then, 10 µm thick sections of the tumor were prepared with a cryomicrotome (CM1900, Leica Microsystems) and exposed to imaging plates (BAS-SR, Fuji Photo Film) for 10 days. Autoradiograms of these sections were obtained with a BAS5000 scanner (Fuji Photo Film).

#### 9.5. Immunohistochemistry and Oil red O staining

Adjacent sections taken from the autoradiographic study were subjected to immunohistochemical staining for FABP4 with hematoxylin counterstaining. A FABP4–specific polyclonal goat IgG (AF3150, Cell Signaling Technology) and perilipin-specific monoclonal rabbit IgG (D1D8, Cell Signaling Technology) were used as the primary antibodies. Histofine Simple Stain MAX-PO (G) (NICHIREI BIOSCIENCES, INC.) and the Envision+ kit (K4002, Dako) were used as the secondary antibodies. Subclass-matched irrelevant IgG served as a negative control. Oil Red O staining was performed by standard procedures.

#### 9.6. Western blotting analysis

In addition to the FABP4 recombinant protein (positive control), whole protein extracts (10 µg) from cultured C6 cells and mouse and rat adipose tissue, as well as excised C6 tumors from tumor-bearing mice were subjected to 5–20% SDS–polyacrylamide gel electrophoresis (E-T520L, ATTO) followed by protein transfer to PVDF membranes. For FABP4 immunological detection, a FABP4–specific monoclonal rabbit IgG (D25B3, Cell Signaling Technology, Inc.) was used with goat anti-rabbit IgG (#7074, Cell Signaling Technology, Inc.) as the secondary antibody. Detection was achieved using Chemi-Lumi One Super (Nacalai Tesque, Inc.). Blots were also incubated with a monoclonal antibody raised against β-actin, which was used as a loading control. The size of the detected proteins was estimated using Kaleidoscope Prestained Standards (Bio-Rad Laboratories, Inc.).

### 10. Statistical analysis

The *in vitro* data, with the exception of those for the cell uptake study, are expressed as mean ± standard errors of the means (SEM), while the data for the cell uptake and *in vivo* studies are expressed as mean ± SD. Statistical analysis was performed with the Mann-Whitney *U* test. *P* values<0.05 were considered statistically significant.

## Results

### 1. *In vitro* study using TAP1

#### 1.1. Binding assay

The affinity of TAP1 for FABP4 was determined in a competition binding assay using the established FABP4 ligand 1,8-ANS. Analysis of inhibition curves resulted in a TAP1 inhibitory constant of *K*_i_ = 44.5±9.8 nM (n = 9).

#### 1.2. Parallel artificial membrane permeation assay (PAMPA)

The permeability of TAP1 was determined by a PAMPA to be 12.6±2.9×10^−6^ cm/s.

### 2. Radiosynthesis

**Figure 1:**
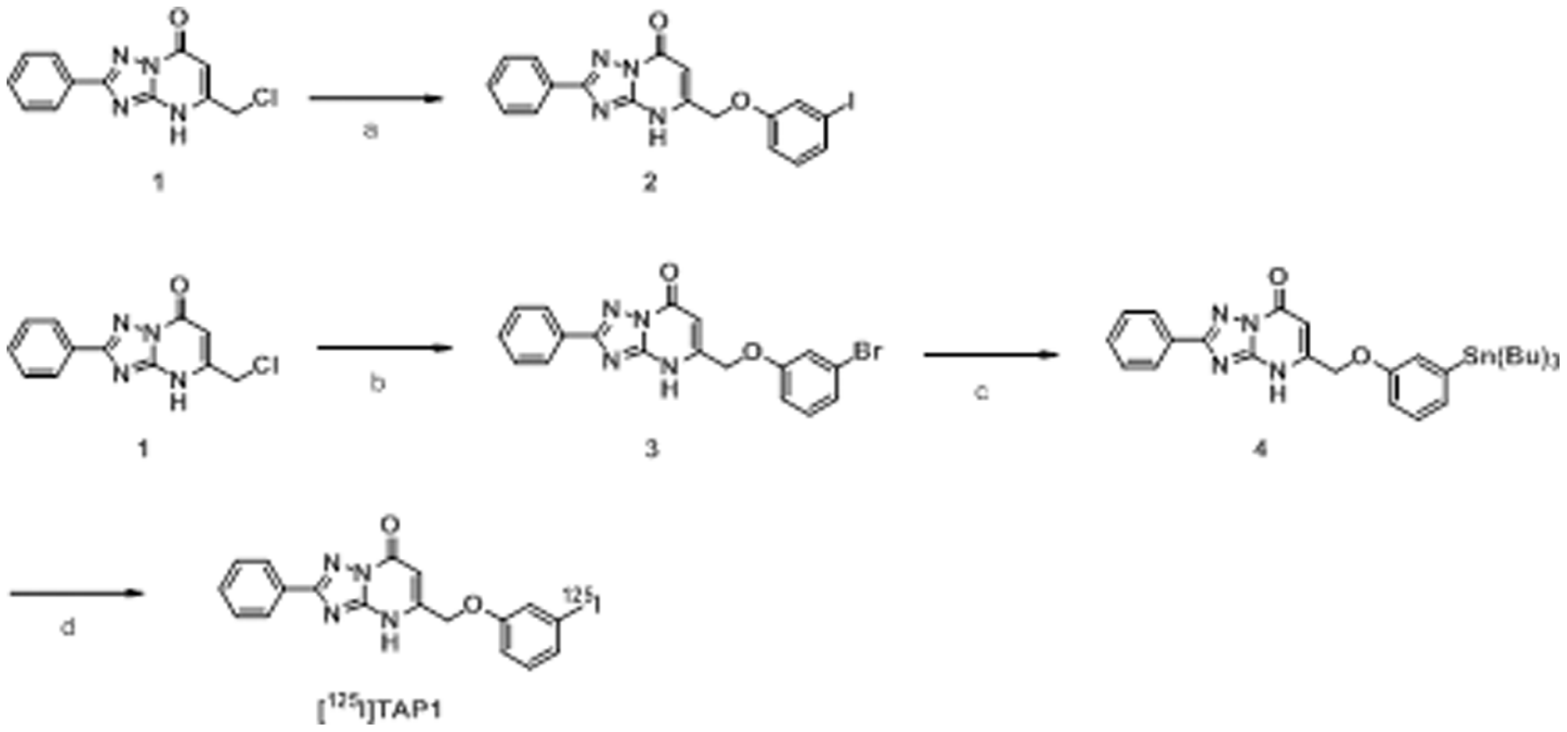
Reagents and conditions. (a) 3-iodophenol, K_2_CO_3_, DMF. (b) 3-bromophenol, K_2_CO_3_, DMF. (c) (Bu_3_Sn)_2_, (Ph_3_P)_4_Pd, Et_3_N, DMF. (d) [^125^I]NaI, NCS, MeOH (1% acetic acid).

The radiosynthesis of [^125^I]TAP1 was accomplished following the reaction sequence shown in Fig. 1. The radiochemical yield was 22.9±0.3%, and the radiochemical purity was above 99% after purification by RP-HPLC. The no-carrier-added preparation was anticipated to result in a final product bearing a theoretical specific activity similar to that of ^125^I (81.4 TBq/mmol).

### 3. *In vitro* study using [^125^I]TAP1

#### 3.1. Binding assay

**Figure 2:**
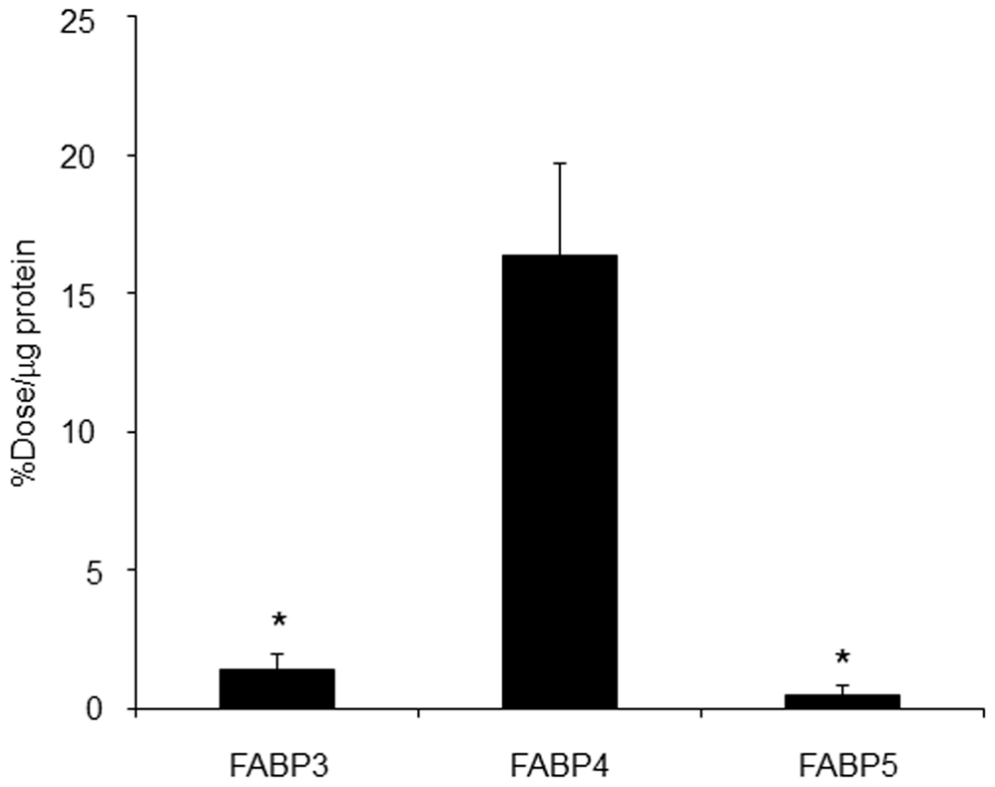
Binding of [^125^I]TAP1 to FABP3, 4, and 5. ^*^ *P*<0.05 vs. FABP4 group.

**Figure 3:**
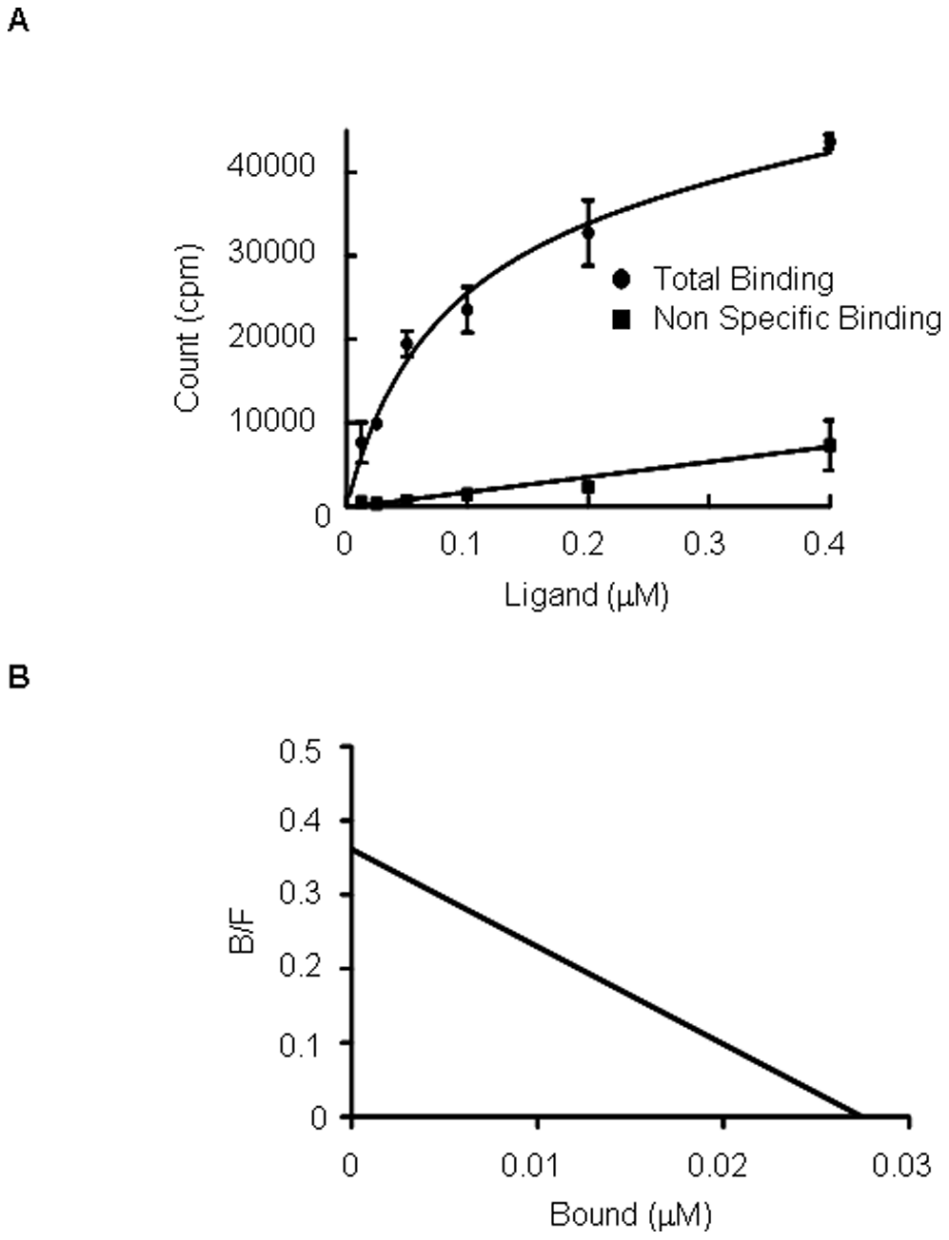
Binding saturation assay of [^125^I]TAP1. (A) Saturation curve of [^125^I]TAP1 for FABP4. (B) Scatchard plots of [^125^I]TAP1 binding to FABP4.

The selectivity of [^125^I]TAP1 towards FABP3 and FABP5 was determined using recombinant proteins and Ni-NTA Magnetic Agarose Beads (Fig. 2). [^125^I]TAP1 binding to FABP4 was 11.5-fold higher than to FABP3 and 33.2-fold higher than to FABP5 (16.3±3.4% dose/µg protein (FABP4) vs. 1.4±0.5% dose/µg protein (FABP3) and 0.5±0.3% dose/µg protein (FABP5), *P*<0.05). For determination of the dissociation constant of [^125^I]TAP1 to FABP4, a saturation binding assay with [^125^I]TAP1 was performed (Fig. 3). The FABP4 binding of [^125^I]TAP1 was found to be saturable while Scatchard transformation of saturation binding data gave a linear plot, suggesting that there is a single high-affinity binding site with a *K*_d_ of 69.1±12.3 nM.

#### 3.2. Cell uptake study

**Figure 4:**
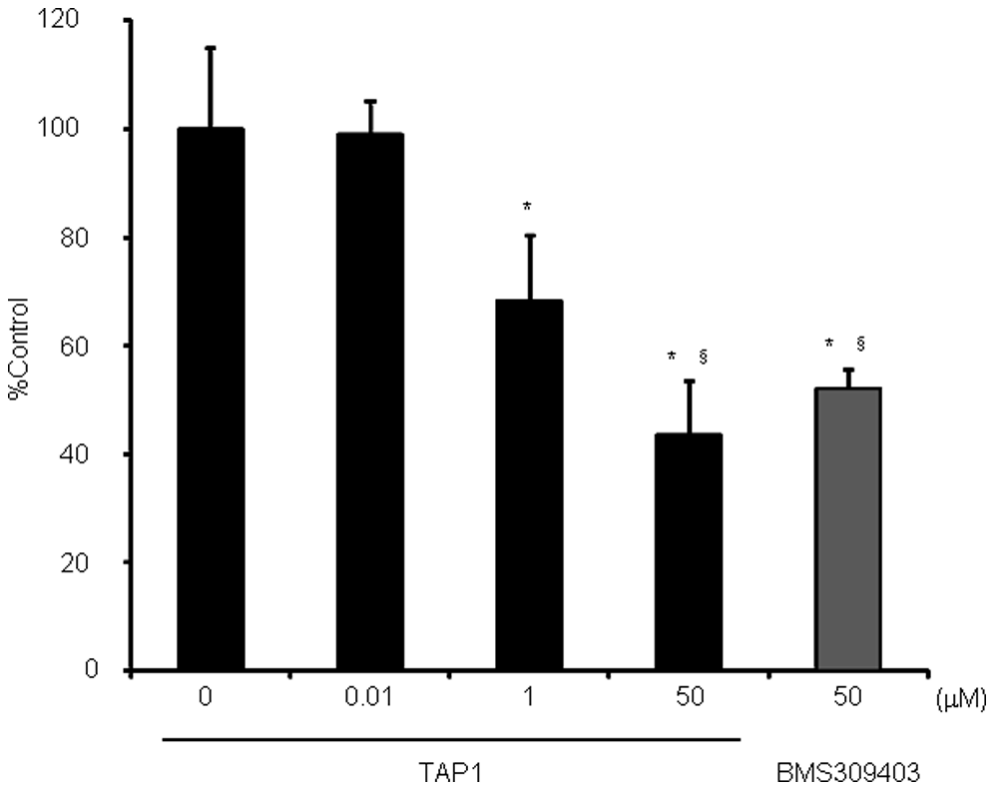
Uptake of [^125^I]TAP1 into differentiated THP-1 cells and inhibition by TAP1 or BMS309403. *P*<0.05 vs. 0 µM group, ^§^ *P*<0.05 vs. 0.01 µM.

Further investigation of [^125^I]TAP1 uptake into differentiated THP-1 cells was performed to clarify whether [^125^I]TAP1 binds to intracellular FABP4 (Fig. 4). [^125^I]TAP1 uptake into differentiated THP-1 cells was significantly inhibited by nonradioactive TAP1 in a concentration dependent manner and was significantly inhibited by the FABP4 inhibitor BMS309403. In addition, in a study using 3T3-L1 cells and differentiated adipocytes, FABP4 was found to be expressed in differentiated adipocytes, but not in 3T3-L1 cells (Fig. S1A). The amount of [^125^I]TAP1 taken into adipocytes (FABP4 positive) was significantly higher than that by 3T3-L1 preadipocytes (FABP4 negative). This uptake could be significantly inhibited by the FABP4 inhibitor BMS309403 in a concentration-dependent manner (Fig. S1B).

#### 3.3. Determination of partition coefficient, stability, and serum protein binding

**Figure 5:**
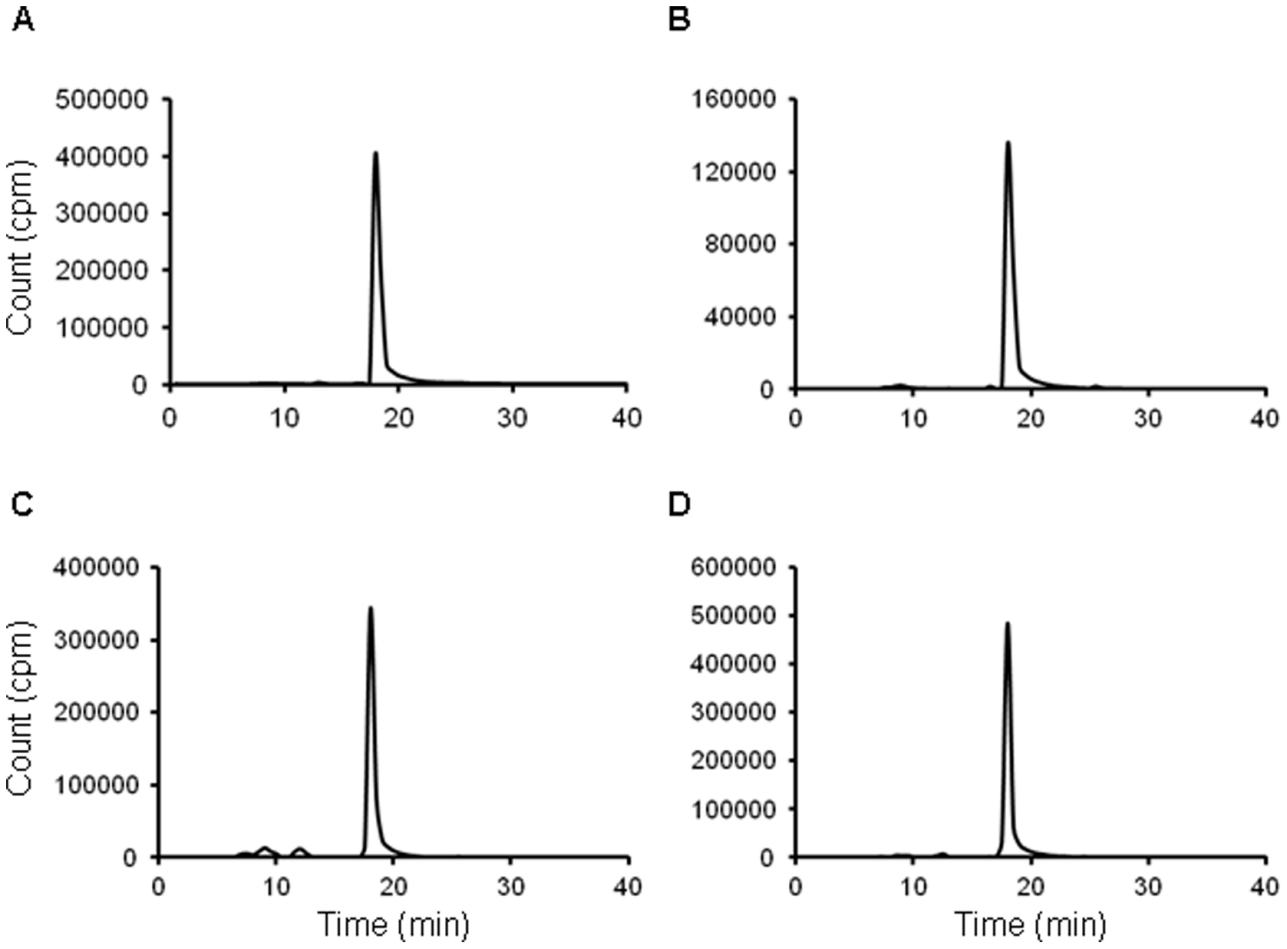
*In vitro* stability estimation and *ex vivo* metabolite analysis. HPLC radiochromatograms of [^125^I]TAP1 after a 2 hr incubation at 37 °C in (A) saline and (B) mouse plasma. HPLC radiochromatograms of radioactivity extracted from (C) liver and (D) kidneys 1 hr after intravenous administration of [^125^I]TAP1 in normal mice. The main peaks observed at 18 min in the four chromatograms correspond to the intact form of [^125^I]TAP1.

Measurement of the [^125^I]TAP1 partition coefficient resulted in a log*P* value of 2.7±0.1. After incubation of [^125^I]TAP1 in saline (Fig. 5A) and mouse plasma (Fig. 5B) for 2 hr at 37 °C, no radioactive degradation products were observed in HPLC analysis, thus demonstrating that [^125^I]TAP1 was highly stable in plasma. The percentage of [^125^I]TAP1 bound to serum proteins was found to be more than 99%.

### 4. *In vivo* and *ex vivo* studies using [^125^I]TAP1

#### 4.1. Biodistribution in normal mice

The biodistribution of [^125^I]TAP1 was next evaluated in normal mice. As shown in Table 1, [^125^I]TAP1 was rapidly cleared mainly through excretion via the liver while low level radioactivity was observed in the stomach and thyroid gland, suggesting that [^125^I]TAP1 was stable against deiodination *in vivo*.

**Table 1:** Biodistribution of radioactivity after intravenous administration of [^125^I]TAP1 in normal mice.

	Time after injection (min)				
	5	15	30	60	120
Blood	3.25±0.20	3.91±0.75	3.45±0.41	2.02±0.35	1.72±0.49
Heart	1.59±0.54	1.57±0.51	1.39±0.25	0.96±0.32	1.11±0.23
Lung	3.12±1.11	2.88±0.65	2.76±0.49	1.93±0.91	1.46±0.13
Liver	57.51±4.79	47.58±2.57	50.91±7.27	46.70±4.43	31.00±1.52
Kidney	15.30±9.93	32.89±11.78	45.34±3.34	45.89±13.85	50.84±18.75
Intestine	2.21±1.43	2.21±0.72	3.83±0.07	5.10±1.43	10.58±1.93
Stomach^a^	0.40±0.14	0.76±0.14	0.92±0.21	1.40±0.69	2.02±0.70
Spleen	1.54±0.60	1.84±0.43	1.29±0.13	1.14±0.38	1.44±1.21
Pancreas	1.53±0.84	1.23±0.26	0.89±0.12	1.46±0.55	0.64±0.1
Muscle	0.73±0.51	0.83±0.09	0.67±0.16	0.64±0.20	0.71±0.24
Thyroid gland^a^	0.01±0.005	0.03±0.01	0.03±0.01	0.02±0.08	0.14±0.21

Data are presented as % injected dose per gram. Each value represents the mean±S.D. for 3 animals at each interval.

a Presented as % injected dose per organ.

#### 4.2. Metabolite analysis

One hr after [^125^I]TAP1 administration, the radioactive chemical composition in the liver (Fig. 5C) and kidneys (Fig. 5D) was analyzed by RP-HPLC. With both organs, more than 90% of [^125^I]TAP1 remained intact.

#### 4.3. Biodistribution in C6 bearing mice

[^125^I]TAP1 biodistribution was also evaluated in tumor-bearing mice. Because [^125^I]TAP1 blood clearance was revealed to be relatively slow in a study using normal mice (Table 1), the accumulation of [^125^I]TAP1 in each organ was evaluated until 3 hr after the injection in the biodistribution study using C6-bearing mice. As shown in Table 2, levels of radioactivity accumulation and elimination in each organ were the same as for normal mice. [^125^I]TAP1 showed moderate radioactivity accumulation in tumors, which peaked 15 min after injection (2.26±0.45%ID/g). This time is longer than for most other organs examined (with the exception of excretory organs such as the liver, kidney and intestine) that showed accumulation peaks 5 min after injection and were retained during the experimental time window. The tumor to muscle ratio of radioactivity accumulation increased in a time-dependent manner and reached 3.6 at 3 hr after probe injection. To evaluate the influence of radioactivity in any blood that remained in the tumor, the radioactivity accumulation in tumor tissues of mice sacrificed by transcardial perfusion and decapitation was compared. This examination was performed at 3 hr after the injection because tumor to muscle and tumor to blood ratios were maximum at this time point. There was no difference in tumor accumulations (0.96±0.18%ID/g vs. 0.85±0.18%ID/g).

**Table 2:** Radioactivity biodistribution after intravenous administration of [^125^I]TAP1 in C6 bearing mice.

	Time after injection (min)				
	5	15	30	60	180
Blood	10.40±2.34	6.60±0.61	4.74±1.17	3.25±0.66	3.29±0.40
Heart	3.36±0.64	2.45±0.41	1.76±0.40	1.12±0.17	1.14±0.04
Lung	6.76±1.12	4.26±0.19	3.16±1.07	2.19±0.52	1.88±0.25
Liver	46.01±5.99	37.58±2.88	42.20±1.51	30.46±3.28	33.97±4.98
Kidney	29.66±2.24	62.82±9.70	77.74±8.14	71.54±8.25	73.44±4.42
Intestine	2.35±0.34	5.08±0.95	7.97±1.32	11.59±0.20	15.76±3.03
Stomach^a^	0.42±0.16	1.02±0.38	0.60±0.13	0.73±0.40	0.89±0.40
Spleen	2.20± 0.44	1.09±0.10	0.84±0.14	0.65±0.08	0.82±0.11
Pancreas	2.55±0.31	1.20±0.20	0.88±0.13	0.63±0.08	0.70±0.13
Muscle	1.05±0.33	0.74±0.15	0.57±0.12	0.37±0.05	0.39±0.09
Tumor	1.94±0.44	2.26±0.45	1.63±0.38	1.09±0.05	1.37±0.24
T/M ratio	1.94±0.52	3.08±0.10	2.96±0.73	2.95±0.41	3.60±0.98
T/B ratio	0.19±0.04	0.34±0.06	0.35±0.03	0.34±0.05	0.42±0.04

Data are presented as % injected dose per gram. Each value represents the mean ± S.D. for 3 animals at each interval.

a Presented as % injected dose per organ.

#### 4.4. *Ex vivo* autoradiography, immunohistochemistry and western blotting analysis

**Figure 6:**
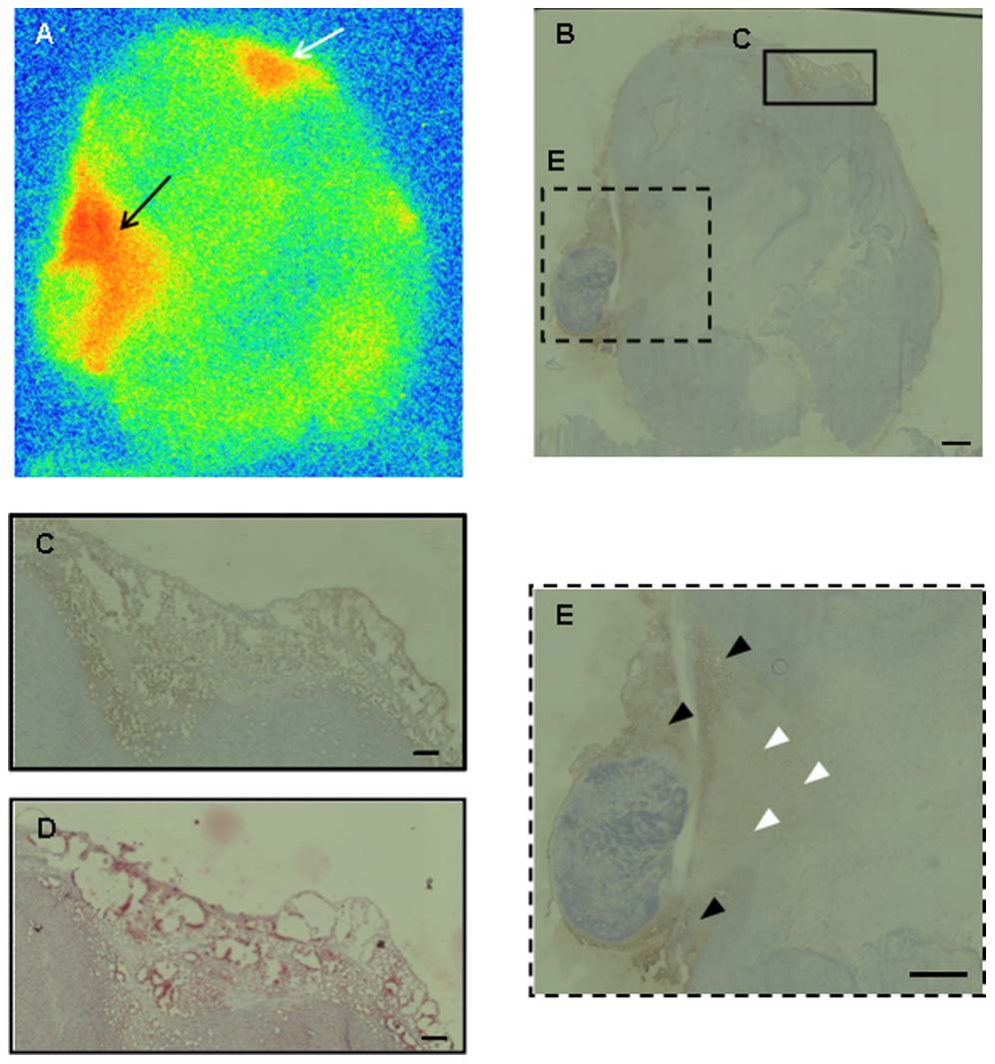
Regional distribution of radioactivity after [^125^I]TAP1 injection and FABP4 expression in tumor sections. Autoradiogram (A) and immunohistochemical staining (B, C, E) of tumor sections. The frames in (B) show the location of the areas captured at higher magnification in C (solid line) and E (dashed line). (D) Oil Red staining of the same area as C. Bar = 1 mm (B, E) and 200 µm (C, D)

**Figure 7:**
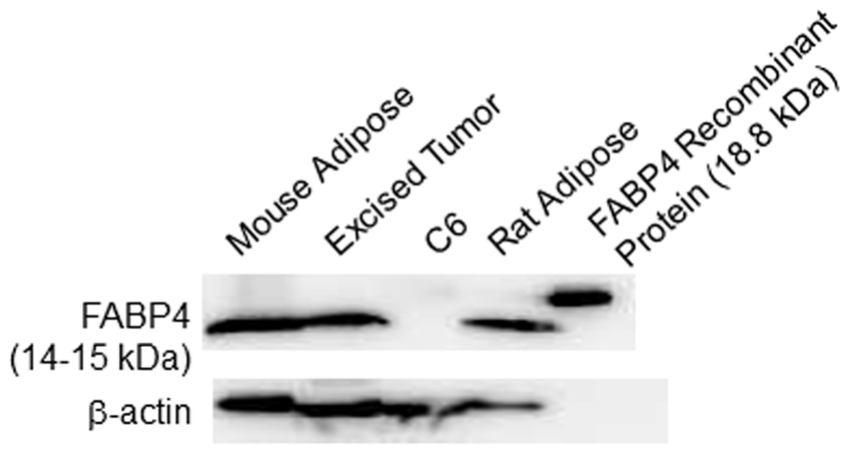
Western blotting analyses of FABP4 and β-actin expression in mouse adipose tissue, excised tumor, C6 cultured cells and rat adipose tissue.

As shown in Fig. 6, a heterogeneous radioactivity distribution was observed in tumors excised 3 hr after [^125^I]TAP1 injection (Fig. 6A). The radioactivity accumulation profile corresponded to the FABP4-positive area in the solid box shown in Fig. 6B (white arrow in Fig. 6A and magnified in Fig. 6C) but did not perfectly match with that in the dashed box shown in Fig. 6B (black arrow in Fig. 6A and magnified in Fig. 6E). In the magnified area shown in Fig. 6E, rather broad radioactivity accumulation was observed (Fig. 6A) in comparison with FABP4 expression (black arrowheads in Fig. 6E), indicating that probe delivery might be affected by tumor perfusion (white arrowheads in Fig. 6E). The FABP4-positive area was also stained by Oil Red O, a marker of adipocytes (Fig. 6D). FABP4 expression in the C6 tumor cells was not confirmed. On the other hand, a western blotting study revealed that FABP4 is expressed in excised C6 tumors and mouse and rat adipose tissue, but not in cultured C6 cells (Fig. 7). This result supports the immunohistochemistry findings.

## Discussion

In the present study, we aimed to develop a novel imaging probe to target FABP4. To develop a FABP4 radiolabeled probe, we designed and synthesized [^125^I]TAP1 and evaluated its usefulness with both *in vitro* and *in vivo* tests. [^125^I]TAP1 showed high affinity and selectivity for FABP4 and high uptake into differentiated THP-1 cells and adipocytes expressing FABP4 due to its appropriate physicochemical properties such as membrane permeability and lipophilicity. Beyond its high *in vivo* stability, [^125^I]TAP1 also had preferable biodistribution in normal mice, and moderate tumor accumulation that partly corresponded to the FABP4 *in vivo* expression profile. While some of the [^125^I]TAP1 distribution in tumors would be predominated by perfusion as revealed by *ex vivo* analysis, our data strongly suggest that [^125^I]TAP1 could detect FABP4 *in vitro*, which, to the best of our knowledge, represents the first report on the development of an effective FABP4 targeting probe. [^125^I]TAP1 is therefore a promising lead compound for further refinement for use in *in vivo* FABP4 imaging.

In this study, a triazolopyrimidine scaffold was utilized for development of the FABP4 radiolabeled probe. Although the FABP4 inhibitor BMS309403 is reported to have high *in vitro* and *in vivo* inhibitory potency and a tritium labeled derivative has been developed for bioassays [4], its high lipophilicity (calculated logP was 5.48) likely would render BMS309403 unsuitable for use as an imaging probe. Initially, we also designed and synthesized compounds based on previous reports for anilide [14], pyrimidine [15], and indole derivatives [16] in addition to triazolopyrimidine derivatives, and a preliminary evaluation of these candidate molecular probes was carried out through *in vitro* experiments. We found that the triazolopyrimidine derivatives showed the highest affinity for FABP4 among the compounds tested, and furthermore, this structure lacked a carboxylic acid group, so that the resulting neutral compound would likely have high cell membrane permeability. Thus, we further evaluated the potential of the radiolabeled triazolopyrimidine derivative, [^125^I]TAP1, as a FABP4 imaging probe. An *in vitro* assay revealed the high affinity of TAP1 for FABP4 that was comparable to BMS309403 (the *K*_i_ values of TAP1 and BMS309403 were 44.5±9.8 and 17.1±1.5 nM, respectively) and moderate lipophilicity, which was reflected in successful intracellular accumulation of [^125^I]TAP1 that could be inhibited by non-radioactive TAP1 and BMS309403.

While [^125^I]TAP1 likely enters cells by passive diffusion and binds to intracellular FABP4 specifically, the transport mechanism for [^125^I]TAP1 cellular uptake remains unclear. Since cellular uptake results suggested that there were differences between the probe uptake levels and background (nonspecific) accumulation levels among the cells, a detailed evaluation of the transport mechanism would help define several characteristics of [^125^I]TAP1. In addition, FABP4 expression levels could vary between cell types. Both differentiated THP-1 cells and adipocytes were used as FABP4-positive model cell types in this study, so determination of the uptake and expression level of FABP4 in various types of cells would further clarify the relationship between *in vitro* uptake and *in vivo* accumulation of [^125^I]TAP1.

Regarding applications of FABP4 molecular imaging, several diseases can be considered as imaging targets for diagnosis. For glioma imaging, radiolabeled amino acids such as [^11^C]methyl-L-methionine (^11^C-MET), O-(2-^18^F-fluoroethyl)-L-tyrosine (^18^F-FET) and 3′-deoxy-3′-[^18^F]-fluoro-L-thymidine (FLT) are currently widely used. While these probes are readily taken up by glioma tissues due to the activation of amino acid transporters or DNA synthesis, the accumulation levels of these probes do not necessarily correlate with malignancy, i.e., WHO glioma grades [17]. The finding that FABP4 is only expressed in tumor stromal cells and not tumor cells [7], could distinguish FABP4 targeting probes from the probes that are currently in use. In this study, we confirmed by immunohistochemical and western blotting analyses that FABP4 was indeed only expressed in tumor stroma and not in tumor cells. Thus, [^125^I]TAP1 would be a unique probe that has the potential to evaluate tumor stromal functions. In addition, in other tumors, such as bladder cancer, FABP4 was reported to be expressed in tumor cells and is related to the pathology [18], [19]. As such, [^125^I]TAP1 could have applications for clarifying the relationship between tumor malignancy and the function of FABP4 in such tumors.

In atherosclerosis, FABP4 expression was reported to correlate with plaque instability in a study using endarterectomy samples collected from patients with symptomatic and asymptomatic carotid stenosis [20]. While 2-^18^F-fluoro-2-deoxy-D-glucose (^18^F-FDG) is considered to be a useful radio-probe for evaluating plaque characteristics [21], this probe also accumulates in macrophages that range from relatively stable to atheromatous in atherosclerotic lesions [22]. On the other hand, since FABP4 expression is reportedly elevated in activated macrophages with oxidized low density lipoprotein (OxLDL) through activation of nuclear factor κB and protein kinase C [23], a FABP4 targeting probe may be preferable for specifically detecting unstable plaques. In fact, in addition to ^18^F-FDG, many researchers, including our group, have reported atherosclerosis imaging probes that target a variety of cells and functional molecules such as apoptotic cells [24], αvβ3/αvβ5 integrin [25], membrane type 1 matrix metalloproteinase [26], OxLDL [27], lectin-like oxidized low-density lipoprotein receptor 1 [28] and tissue factor [29]. So, for the evaluation of the utility of [^125^I]TAP1 in imaging unstable plaques, a comparative study considering the expression levels of a targeted molecule as well as pharmacokinetics and uptake level of the probe would be needed.

For future applications of TAP1 in *in vivo* molecular imaging, isoform selectivity and lipophilicity may be issues for consideration. For isoform selectivity, TAP1 showed a more than 10-fold higher affinity for FABP4 than FABP3 and FABP5. However, the affinity of BMS309403 for FABP4 is reported to be 300-fold greater than its FABP5 affinity [30]. While high selectivity of molecular probes would be essential for targeting a biomolecule isoform that has a variety of functions and exists at comparable expression levels in close proximity to other isoforms, for FABP4 selectivity may not be as high a priority given that this isoform is expressed in lesions such as glioblastoma and atherosclerosis, while other FABP isoforms are expressed in surrounding organs. Given these factors, the selectivity level required for *in vivo* FABP4 imaging should be clarified using animal disease models.

For lipophilicity, the data showing that [^125^I]TAP1 uptake into THP-1 cells and adipocytes was not fully inhibited in the presence of excess TAP1 or BMS309403 suggests that there may be nonspecific accumulation of [^125^I]TAP1 in these cells. This was also implied from the high serum protein binding ratio, which would have an effect on the pharmacokinetics of [^125^I]TAP1 and in fact cause non-specific accumulation in tumors due to perfusion that is beyond the ‘true’ distribution that corresponds to FABP4 expression. As mentioned above, moderate lipophilicity is necessary for intracellular targeting, and as such a thorough investigation to identify an optimal lipophilicity range might be needed. To reduce the lipophilicity to within the optimal range, replacement of radioiodine with other radiohalogens such as radiofluorine (^18^F) and radiobromine (^76/77^Br) and introduction of some hydrophilic groups such as a hydroxyl group would be possible approaches [31]. [^123^I]TAP1 is a SPECT probe that takes advantage of the high versatility of SPECT and recent improvements in the spatial resolution provided by this technique. Although the substitution of ^123^I for ^76^Br or ^18^F would require a change in imaging modality to positron emission tomography (PET), which has the advantage of spatial resolution and quantitativity, both SPECT and PET have merits and as such changes would not be a problem at this stage. Therefore, labeled radionuclide substitution could be an option to refine probe characteristics for future studies.

FABP4 imaging could be useful for diagnosing glioma malignancy and instability of atherosclerotic plaques, to elucidate the detailed function of FABP4 in FABP4-related diseases, and also to develop pharmaceuticals that target FABP4 in these disorders. In this study, [^125^I]TAP1 was demonstrated to specifically detect FABP4 *in vitro* and partly *in vivo*. Although FABP4 expressed in cells surrounding the tumor such as adipocytes and macrophages plays an important role in the metabolic microenvironment of glioma and other tumors [7], [32], we should stress here that the detailed function of FABP4 still remains unclear. As such, an imaging probe targeting FABP4 could also be useful for elucidating how FABP4 functions in glioma and related diseases. In any case, although further modifications are required, [^125^I]TAP1 might be a potential lead compound for developing radiolabeled probes for use in *in vivo* FABP4 imaging.

In conclusion, we developed a novel radiolabeled probe, [^125^I]TAP1, which showed specificity and selectivity for FABP4, suitable *in vitro* physicochemical characteristics, and partial validity in *in vivo* assays. Further probe refinements toward an effective FABP4 imaging probe and additional investigations using various animal disease models are warranted.

## Supporting Information

Figure S1 **Western blotting analyses and cellular uptake study of [^125^I]TAP1.** (A) Western blotting analyses of FABP4 and β-actin expression in cultured 3T3-L1 cells and adipocytes. (B) Uptake of [^125^I]TAP1 into adipocytes and 3T3-L1 cells, and inhibition by BMS309403. ^#^ *P*<0.05 vs. 3T3-L1 group, ^†^ *P*<0.05 vs. 1 µM group, ^§^ *P*<0.0001 vs. 10 µM group.
(TIF)

Method S1 **Supplementary materials and methods for western blotting analyses and cellular uptake study of [^125^I]TAP1.**
(DOCX)
