# Supplementary material for: Development of a Radioiodinated Triazolopyrimidine Probe for Nuclear Medical Imaging of Fatty Acid Binding Protein 4
Source: PLoS One. 2014 Apr 14;9(4):e94668. doi: 10.1371/journal.pone.0094668 (PMC3986099; doi:10.1371/journal.pone.0094668)
Supplement: Method S1 — Supplementary materials and methods for western blotting analyses and cellular uptake study of [125I]TAP1. (DOCX) [file pone.0094668.s002.docx]

**Methods**

*1. Cell culture*

Murine 3T3-L1 preadipocytes were obtained from ATCC and cultured in Dulbecco's Modified Eagle Medium (Nissui Pharmaceutical Co., Ltd.) supplemented with 10% heat-inactivated fetal bovine serum, 50 U/ml penicillin and 50 μg/ml streptomycin at 37 °C in 5% CO_2_. For cell uptake assay, differentiation of 3T3-L1 cells was performed as described previously [1]. Briefly, two days after confluence, the medium was replaced with DMEM containing 10% FBS and inducers (0.25 mM dexamethasone, 10 μg/ml insulin, 0.5 mM 3-isobutyl-1-methylxanthine and 10 μM pioglitazone hydrochloride) for 2 days. The medium was then changed to DMEM supplemented with 10% FBS, 10 μg/ml insulin and 10 μM pioglitazone hydrochloride, and then incubated for 2 days. Then, the medium was changed to DMEM supplemented with 10% FBS and 10 μM pioglitazone hydrochloride every other day. After 8-10 days, the differentiated adipocytes were used for experiments.

*2. Western blotting analysis*

Whole protein extracts (10 μg) from cultured 3T3-L1 cells and adipocytes were subjected to 5–20% SDS–polyacrylamide gel electrophoresis (E-T520L, ATTO) followed by protein transfer to PVDF membranes. For FABP4 immunological detection, a FABP4–specific monoclonal rabbit IgG (D25B3, Cell Signaling Technology, Inc.) was used with goat anti-rabbit IgG (#7074, Cell Signaling Technology, Inc.) as the secondary antibody. Detection was achieved using Chemi-Lumi One Super. Blots were also incubated with a monoclonal antibody raised against β-actin, which was used as a loading control. The size of the detected proteins was estimated using Kaleidoscope Prestained Standards (Bio-Rad Laboratories, Inc.).

*3. Cell uptake study*

For the cell uptake study using adipocytes, the medium of 24-well plate, in which the differentiated adipocytes or undifferentiated 3T3-L1 cells are cultured, was removed. After washing with PBS (-), 0.05 ml BMS309403 in DMEM (0-100 μM, 1% DMSO, v/v) and 0.40 ml DMEM were added and incubated at 37 °C for 1 hr. Then, 0.05 ml [^125^I]TAP1 (0.037 MBq, 10% DMSO, v/v) was added and incubated at 37 °C for 1 hr. After washing with 0.5 ml PBS (-) (1% DMSO and 0.1 % tween 20, v/v) three times, 0.25 ml 2N NaOH was added for cytolysis. Following the measurement of the radioactivity of collected cell lysate with a well-type γ-counter, protein determination was performed. After the cells were lysed with 0.25 ml 2N NaOH, the protein determination was performed. Binding ratios were calculated as (radioactivity in collected cells)/(total applied radioactivity)/(protein abundance) x 100 (%Dose/mg protein).

**Reference**

1. Kimura I, Ozawa K, Inoue D, Imamura T, Kimura K, et al. (2013) The gut microbiota suppresses insulin-mediated fat accumulation via the short-chain fatty acid receptor GPR43. Nature Communications 4: 1829.
